# HIPPI: highly accurate protein family classification with ensembles of HMMs

**DOI:** 10.1186/s12864-016-3097-0

**Published:** 2016-11-11

**Authors:** Nam-phuong Nguyen, Michael Nute, Siavash Mirarab, Tandy Warnow

**Affiliations:** 1 Department of Computer Science and Engineering, University of California, San Diego, 9500 Gilman Drive, La Jolla, 92093 CA USA; 2 Department of Electrical and Computer Engineering, University of California, San Diego, 9500 Gilman Drive, La Jolla, 92093 CA USA; 3 Department of Computer Science, University of Illinois at Urbana-Champaign, 201 North Goodwin Ave, IL, Urbana, 61801 IL USA; 4 Department of Statistics, University of Illinois at Urbana-Champaign, 725 South Wright Street, Urbana, 61820 IL USA; 5 Department of Bioengineering, University of Illinois at Urbana-Champaign, 1270 Digital Computer Laboratory, Urbana, 61801 IL USA; 6 Carl R. Woese Institute for Genomic Biology, University of Illinois at Urbana-Champaign, 1206 West Gregory Drive, Urbana, 61801 IL USA; 7 National Center for Supercomputing Applications, University of Illinois at Urbana-Champaign, 1205 West Clark Street, Urbana, 61801 IL USA

**Keywords:** Protein family identification, Ensemble of profile hidden Markov models, Pfam

## Abstract

**Background:**

Given a new biological sequence, detecting membership in a known family is a basic step in many bioinformatics analyses, with applications to protein structure and function prediction and metagenomic taxon identification and abundance profiling, among others. Yet family identification of sequences that are distantly related to sequences in public databases or that are fragmentary remains one of the more difficult analytical problems in bioinformatics.

**Results:**

We present a new technique for family identification called HIPPI (Hierarchical Profile Hidden Markov Models for Protein family Identification). HIPPI uses a novel technique to represent a multiple sequence alignment for a given protein family or superfamily by an ensemble of profile hidden Markov models computed using HMMER. An evaluation of HIPPI on the Pfam database shows that HIPPI has better overall precision and recall than blastp, HMMER, and pipelines based on HHsearch, and maintains good accuracy even for fragmentary query sequences and for protein families with low average pairwise sequence identity, both conditions where other methods degrade in accuracy.

**Conclusion:**

HIPPI provides accurate protein family identification and is robust to difficult model conditions. Our results, combined with observations from previous studies, show that ensembles of profile Hidden Markov models can better represent multiple sequence alignments than a single profile Hidden Markov model, and thus can improve downstream analyses for various bioinformatic tasks. Further research is needed to determine the best practices for building the ensemble of profile Hidden Markov models. HIPPI is available on GitHub at https://github.com/smirarab/sepp.

**Electronic supplementary material:**

The online version of this article (doi:10.1186/s12864-016-3097-0) contains supplementary material, which is available to authorized users.

## Background

The assignment of newly obtained molecular sequences to gene families or protein families and superfamilies is a fundamental step in many bioinformatics analyses. For example, newly discovered protein sequences are assigned to protein families or superfamilies to enable functional annotation [1, 2]. Similarly, sequences obtained in shotgun sequencing analyses of environmental samples are often assigned to gene families in order to perform marker-based taxonomic identification [3–7]. This assignment is very difficult when the query sequence is very short (a typical problem for transcriptomic and metagenomic datasets), or when the query sequence shares little sequence similarity to any of the sequences in published databases [8]. Therefore, improving the precision and recall of methods that classify sequences into existing families is an area of active research.

Techniques for protein family classification and gene binning operate in two basic steps: first, the query sequence is compared to each family in a published database and the probability of membership in the family is assessed; then, the family with the highest probability is returned for that query sequence, provided the probability is above a required minimum threshold. The first step of this process thus uses tools for homology detection. One of the simplest methods for homology detection is BLAST [9], including variations designed specifically for proteins, such as blastp and PSI-BLAST [10]. While these sequence similarity-based approaches have good accuracy in many conditions, they can have poor accuracy when classifying query sequences that have low sequence similarity to all the sequences in the reference database [11].

A different approach is to represent each family as a single profile hidden Markov model (HMM) and assign the query sequence to the family whose profile HMM returns the highest bit score for the query sequence [12]. For example, this approach is used to assign query sequences to Pfam [13] protein families and superfamilies, where the HMMER [14, 15] software suite is used to build HMMs and assign query sequences to the best fitting Pfam family. Additionally, it has been used in several other applications, including the identification of viral families from metagenomic samples [16].

A related approach is the HHsearch pipeline [11], which is packaged with HHblits [17] in the publicly available HHpred server [18]. This pipeline takes the query sequence and finds a set of homologous sequences from a reference protein database. It then builds an HMM on a multiple sequence alignment of the query sequence and its homologous sequences, aligns the HMM against the individual HMMs built on each of the protein families, and finally assigns the query sequence to the family with the best score. The HHsearch pipeline has been shown to perform well on remote homology detection [19].

Some methods use a collection of profile HMMs (which we call ensembles of HMMs) to represent a protein family, including [3, 20]. Qian and Goldstein [20] expand on the use of tree-HMMs (described originally in [21, 22]) to represent an alignment. These tree-HMMs have a profile HMM on every node in the tree, and are called T-HMMs. Brown et al. [3] describe a two-step process in which the first step uses SCI-PHY to identify subfamilies using an alignment and a tree for a protein family, and then the second step builds subfamily HMMs for the identified subfamilies. Several studies have shown that using an ensemble of HMMs to represent a protein family has resulted in improved protein family identification for remote homologs, improved subfamily identification, and improved orthology detection [3, 20, 23–25].

We have developed similar methods for representing a multiple sequence alignment with an ensemble of HMMs. These methods include SEPP [26], TIPP [6], and UPP [27] (collectively referred to as the ^∗^PP methods). One of the key difference between the ^∗^PP methods and previous ensemble approaches (i.e., [3, 20]) is that the profile HMMs generated by the ^∗^PP methods are based upon a recursive subdivision of an input tree into approximately equally-sized subtrees (referred to as a “centroid decomposition”), resulting in fewer profile HMMs, without requiring the profile HMMs to be based upon clades. Nguyen et al. [27] found that a clade-based decomposition created a more computationally intensive process but did not improve accuracy. The ensembles of HMMs generated by the ^∗^PP methods have been shown to improve the accuracy of phylogenetic placement, taxon identification of short metagenomic reads, and multiple sequence alignment estimation.

**Fig. 1:**
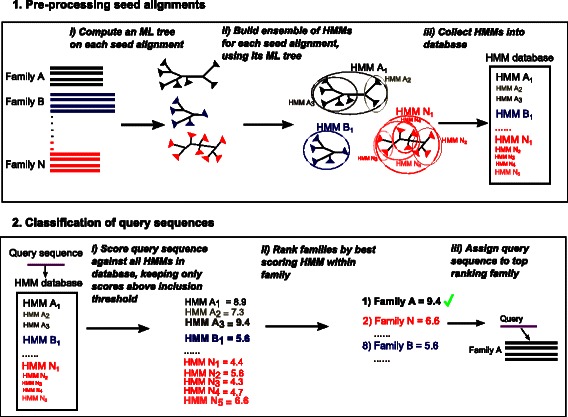
Overview of the HIPPI algorithm. The input is a collection of seed alignments. Box 1 shows the preprocessing phase of building the ensemble of HMMs database from the seed alignments. Box 2 shows the classification phase of using the ensemble of HMMs database to classify the query sequences. See Fig. 2 for details of how the ensemble of HMMs is constructed

In this paper, we present a new method, HIPPI (**HI**erarchical **P**rofile HMMs for **P**rotein family **I**dentification; see Fig. 1 for an overview of the algorithm), that classifies query amino acid sequences into protein families and superfamilies. HIPPI modifies the previous ^∗^PP methods to address the problem of family selection. As in the ^∗^PP methods, HIPPI builds an ensemble of profile HMMs to represent each protein family, but it improves on earlier techniques for building ensembles of profile HMMs by changing the dataset decomposition strategy to take pairwise sequence identity into account. Given a query sequence *q*, HIPPI scores *q* against every profile HMM in every ensemble of profile HMMs built from the protein families. Finally, HIPPI assigns *q* to the protein family whose ensemble of HMMs has the profile HMM that reports the best bit score for the query sequence. This is a general approach that can be applied to any collection of protein families.

**Fig. 2:**
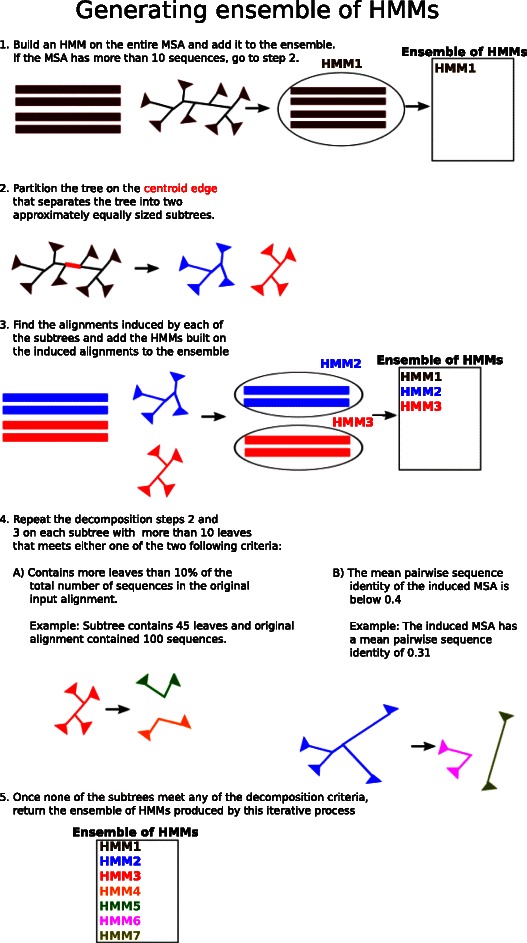
Algorithm for generating the ensemble of HMMs. The input is a seed alignment and a maximum likelihood (ML) tree that has been estimated for the seed alignment. The algorithm begins by adding the HMM built on the seed alignment to the ensemble. If the seed alignment has more than 10 sequences, the ML tree is decomposed into two subtrees by deleting the centroid edge (i.e., the edge that produces a maximally balanced split of the taxon set into two sets). The subtrees are used to generate induced alignments. HMMs are built for each induced alignment and added to the ensemble. The process iterates on those subtrees that meet the criterion for decomposition (subset size more than max(10, n/10), where n is the number of sequences in the seed alignment, and mean pairwise sequence identity less than 40 %)

In our study, we present a comparison between HIPPI, HMMER, blastp, and the HHblits+HHsearch pipeline (referred to herein as “HHsearch”) for the problem of protein family identification using the Pfam-A database of protein families [13]. As we will show, HIPPI provides greater precision and recall than the other methods. Furthermore, HIPPI has substantially better precision and recall than the other methods under the most challenging conditions where the protein family has low average sequence identity and the query sequence is fragmentary.

## HIPPI

The input to the HIPPI algorithm is a database $\mathcal {D} = \{\mathcal {F}_{1}, \mathcal {F}_{2}, \ldots, \mathcal {F}_{k}\}$ of protein families and a query sequence *q*. Every family $\mathcal {F}_{i}$ in the database includes a known (curated) seed alignment. The key feature of HIPPI is in the preprocessing step described below, where the method builds an ensemble of HMMs for each family, based on an estimated maximum likelihood (ML) phylogeny for the family.

### Preprocessing

In the preprocessing step (Box 1 in Fig. 1), we construct an ensemble *H*_*i*_ of HMMs for each protein family $\mathcal {F}_{i}$ in the database $\mathcal {D}$. Here we show how we compute *H*_*i*_, given $\mathcal {F}_{i}$.

1. **Step 1:** We estimate a maximum likelihood tree *T*_*i*_ for the seed alignment for $\mathcal {F}_{i}$, using FastTree-2 [28].
2. **Step 2:** We build the collection of profile HMMs from the seed alignment and ML tree as follows (Fig. 2). Initially, our ensemble of HMMs is empty. Starting with the initial seed alignment, we build a profile HMM on the entire alignment and add the profile to our ensemble of HMMs. Next, if the seed alignment has more than ten sequences, the ML tree is partitioned into two subtrees by removing the centroid edge (i.e., an edge that splits the tree into two subtrees of approximately equal size). For each of the subtrees, we build a profile HMM on the alignment induced by the sequences in the leaf set of the subtree and we add the HMM to the ensemble of HMMs. This decomposition process is applied recursively on those subtrees that have at least ten sequences, until the stopping criterion based upon two parameters, *X* and *Y*, is met: the number of sequences in the subtree is at most *X*% of the initial seed alignment size (referred to as the “maximum decomposition size”) and the average pairwise sequence identity is at least *Y* *%* (referred to as the “minimum average identity threshold”). Therefore, if the initial seed alignment has ten or fewer sequences, the ensemble will only contain the HMM on the initial seed alignment.
  This decomposition process produces a collection of nested profile HMMs, with one profile HMM based upon the entire seed alignment and (potentially) other profile HMMs based on induced alignments with fewer sequences.

This gives us a set of ensembles $\{H_{i}\}_{i=1}^{k}$, where each ensemble *H*_*i*_ is a set of *p*_*i*_ profile HMMs, {*h*_*ij*_,*j*=1,…,*p*_*i*_}.

### Classification

In the homology detection step (Box 2 in Fig. 1), we are given a query sequence *q* and we assign *q* to the family that contains the profile HMM with the highest bit score, provided the bit score is above the chosen threshold for that family. Therefore, if no family produces a bit score for *q* that is above the family’s threshold, then *Family*(*q*) is undefined, and *q* is not assigned to any family.

Formally, the assignment is performed as follows. First, we denote the bit score for sequence *q* given profile HMM *h* by *BS(q,h)*. We let *Family(q)* denote the family to which we assign *q*, and we set

$${} Family(q)= \underset{i=1,\ldots,k }{\mathbf{argmax}} \left[\underset{j=1,\ldots,p_{i}}{\mathbf{max}} \left\lbrace BS(q,h_{ij}) :BS (q,h_{ij})>g_{i} \right\rbrace \right] $$

where *g*_*i*_ is a chosen threshold for family $\mathcal {F}_{i}$ (see below for further discussion on thresholds). If the innermost set in this formula is empty, meaning no family has a bit score above its threshold, then no family is returned.

### Comments

The inclusion of the minimum average identity threshold *Y* differentiates HIPPI from previous ^∗^PP methods, which only considered the size of the subsets in building the ensemble of HMMs. For example, previous ^∗^PP methods either decomposed until each subset was less than 10 % of the full data [26] (roughly producing 20 HMMs in total), or used a fixed decomposition size [27], decomposing until the subsets have at most 10 sequences (typically producing hundreds of HMMs on an alignment with 1,000 sequences). HIPPI, on the other hand, dynamically determines whether additional decompositions are needed based upon the heterogeneity of the subsets.

Finally, we report results using HMMER to build profile HMMs and align sequences to the profiles. However, HIPPI is a general approach for representing an MSA using profile HMMs, and thus can be used with any HMM-based software.

## Performance study

### Data

The Pfam-A database version 28.0 was retrieved and all families were restricted to their curated seed sequences and corresponding alignment. Since the sequences are manually curated with respect to their family assignment, we consider these assignments as de facto ground truth, which enables the cross-validation scheme described below. The set of over 16,000 families was further restricted to those with at least 10 sequences in the seed alignment. This gave us 11,156 families containing a total of 1,238,077 sequences and a diverse distribution of individual family sizes.

Additionally, for each sequence in the test set above, two fragmentary sequences were generated whose lengths were 1/4 and 1/2 of the full-length sequence. The fragments were generated by randomly selecting a contiguous subsequence (i.e., substring) of the desired length from somewhere in the full-length sequence. A link to the data used in this study is provided in HIPPI’s README at [29].

### Cross-validation testing

We conducted a four-fold cross validation test on the Pfam-A dataset as follows. For the seed sequences in each family, 75 % of the seed sequences were retained and used to represent that family (i.e., the training set). The remaining 25 % of the seed sequences were used as query sequences (i.e., the test set). The goal of this experimental design is to examine the accuracy of assigning the query sequences back to its original family using the reduced set of seed alignments to represent each Pfam family. We partitioned the Pfam-A dataset using this scheme four times (i.e., four pairs of test sets and training sets), with each pair of the test and training set corresponding to one cross-fold of the four-fold cross validation. This partitioning scheme used ensures that each seed sequence appears exactly once in each of the cross-folds, with three appearances in the training sets and one appearance in the test set. For very small families, the removal of even one or two sequences can substantially reduce the diversity of the sequences within the family, and thus a minimum family size of 10 was imposed to prevent spurious results on small families. This cutoff was chosen in advance and was not based upon the empirical properties of the Pfam-A dataset.

In each case, every sequence in the test set and each of its two corresponding fragmentary sequences were treated as *de novo* query sequences, and the remaining 75 % was treated as the database of known families for which each query sequence could be determined to be homologous.

**Fig. 3:**
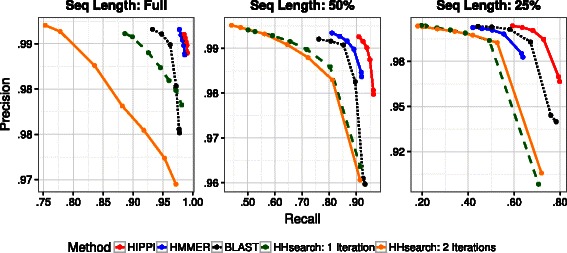
Precision-recall curves for the five methods, evaluated on one cross-fold subset of the data. We vary the length of the query sequence from unchanged (i.e., full) to half-length and quarter-length. The curves are estimated by varying an inclusion threshold parameter for the particular method and producing five to seven distinct points, with intermediate values interpolated linearly. Note that the scales for both axes vary between panels due to the significant impact of sequence fragmentation

**Fig. 4:**
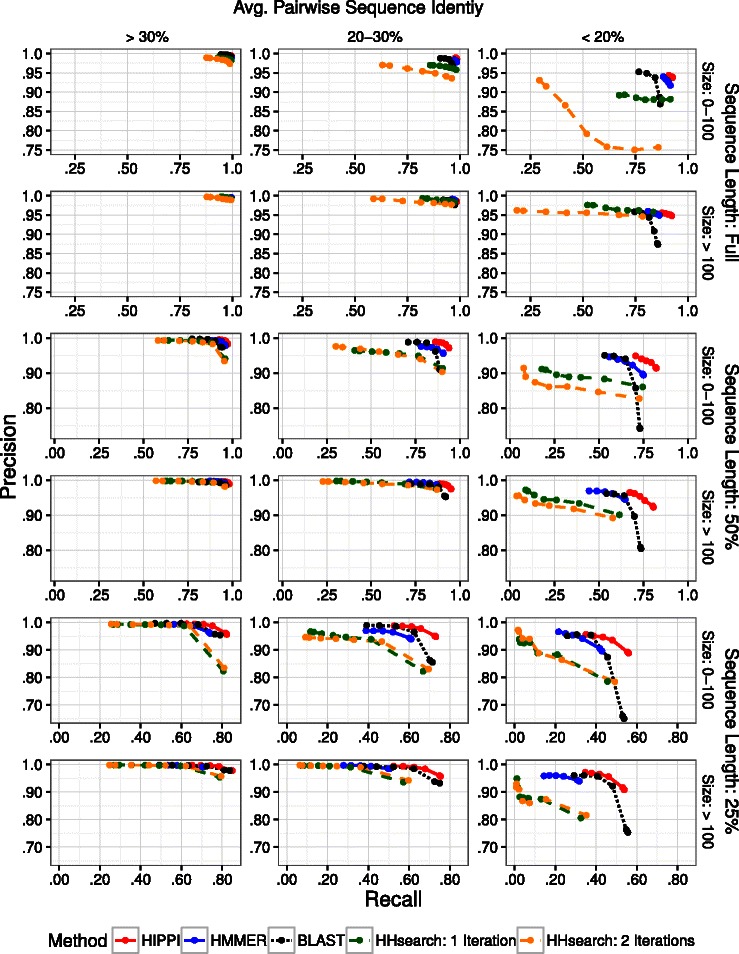
Precision-recall curves for the five methods across different model conditions, evaluated on one cross-fold subset of the data. The columns represent the mean sequence identity of the family, the rows are first grouped by sequence length, and are further subdivided by family size. The curves are estimated by varying an inclusion threshold parameter for the particular method and producing five to seven distinct points, with intermediate values interpolated linearly. Note that the scales for both axes vary between panels due to the significant impacts of segment fragmentation, family size, and sequence identity

The computational requirements for HHsearch (see Running time in “Results and discussion” Section) meant that we could only evaluate HHsearch on one of the four cross-fold subsets. However, we tested HIPPI, HMMER, and blastp on all four cross-fold subsets; the combined results of the all cross-folds are shown in Additional file 1: Figure A2. The performance of these three methods across the different cross-folds is very close - no individual precision or recall for a fold was different from any other by more than 0.2 %. Our main analysis, shown in Figs. 3 and 4, contains the results of all methods tested on just one cross-fold.

## Methods

The implementation of each method on this data is described below. Note that when a method assigns a sequence to a family, it is accompanied by a method-specific score; as a result, the assignment can be rejected if the score is below an inclusion threshold (see below for more details) for that combination of method and dataset. The commands used to replicate the results are given in Additional file 1.

### HMMER

The HMMER pipeline was implemented using HMMER v3.1b2 [14, 15]. An HMM was built on the curated Pfam alignment for the seed sequences of each family in the training data using the HMMER *hmmbuild* command using the default mode. For a given query sequence, the sequence is aligned to the HMM for each family using the HMMER *hmmsearch* command and the family with the highest bit score that is above the inclusion threshold is returned.

### HIPPI

The preprocessing step for HIPPI was run on the training set to build ensembles of HMMs for each family (see Box 1 in Fig. 1), and the classification step was run on every query sequence in the test set (see Box 2 in Fig. 1). We report the family with the highest bit score that is above the inclusion threshold for each query sequence. HIPPI’s performance is impacted by the choices of maximum decomposition size *X* (expressed as a percentage of the full set of sequences) and minimum average identity threshold *Y* (also expressed as a percentage) used in the stopping criterion. A preliminary analysis on a subset of the data (see Table 1 and discussion in HIPPI parameter selection) suggested a default setting of *X*=10 and *Y*=40 (decompose until each subset contains at most 10 % of the initial seed sequences and has an average pairwise sequence identity that is at least 40 %, or the subset contains 10 or fewer sequences). We call this default HIPPI, and results presented for HIPPI in comparison to HMMER, blastp, and HHsearch are under this parameter setting. Note that HIPPI run using a single HMM in the ensemble is equivalent to running HMMER, as described in the preceding paragraph.

**Table 1:** Average precision/recall scores on the 143 Pfam families from the parameter selection dataset across all four-folds for the three different fragment lengths

Method	Fragment Size					
	Quarter-length		Half-length		Full-length	
	Prec.	Rec.	Prec.	Rec.	Prec.	Rec.
HIPPI-c (5 %, 0 %)	92.6 %	33.8 %	86.4 %	47.7 %	85.6 %	79.2 %
HIPPI-c (10 %, 0 %)	**93.1 %**	**34.3 %**	**86.6 %**	45.1 %	85.1 %	78.0 %
HIPPI-c (10 %, 40 %)	92.2 %	33.4 %	**86.6 %**	**48.8 %**	**85.8 %**	**79.5 %**
HIPPI-a (5 %, 0 %)	82.6 %	39.9 %	78.6 %	67.4 %	84.1 %	83.3 %
HIPPI-a (10 %, 0 %)	**82.8 %**	37.8 %	78.1 %	65.2 %	83.6 %	82.7 %
HIPPI-a (10 %, 40 %)	82.5 %	**40.6 %**	**79.3 %**	**68.8 %**	**84.6 %**	**83.8 %**

The methods are labeled as HIPPI-x (*X*%,*Y*%), where “-x” is either “-c” (the conservative setting that uses the default gathering cutoff threshold for the inclusion threshold) or “-a” (the aggressive setting that uses no inclusion threshold), *X*% denotes the maximum decomposition size, expressed as a percentage of the number of sequences in the full dataset, and *Y*% denotes the minimum average pairwise sequence identity within each subset, expressed as a percentage. The best average precision and recall for the conservative and aggressive versions are bolded

### HHsearch

This pipeline was designed to emulate the HHpred server and uses the HH-suite software tools version 3.0.0. For each family in the training set, HHmake was used to generate an HMM on the seed alignment in the training dataset. HHblits was used with the pre-filtered version of the Uniprot20 [30] database, which is the database recommended by the software developers. For every query sequence, an HHblits search was conducted against the Uniprot20 data to generate a multiple sequence alignment. The multiple sequence alignment was scored against the HMMs built on the families in the training set using HHsearch.

The default setting for HHsearch is to use a local alignment and two iterations of HHblits to gather homologs. In order to ensure that the optimal parameters for this pipeline were used, we explored variants where we replaced local alignment by global alignment and used one iteration of HHblits instead of two. We tested these alternative settings on 30,000 sequences from the holdout data to understand their impact, particularly on fragmentary sequences. 10,000 of the sequences were randomly selected from the full-length set; we then included the half-length and quarter-length versions of the selected full-length sequences, for a total of 30,000 sequences. We label the default setting by “HHsearch: 2 Iterations”. We began by comparing HHsearch: 2 Iterations using global alignment to the default setting; this comparison showed that for all sequence lengths studied, the local alignment strategy produced better results. We then compared the use of one iteration to two iterations of HHblits, followed by local alignment. The results of these initial tests are provided in Additional file 1: Figure A1. No single way of running HHsearch provided the best accuracy across all the conditions explored, but the two best methods both use local alignment. Hence, we show the two top performing settings, HHsearch: 1 Iteration and HHsearch: 2 Iterations, on the holdout data; see “Results and discussion” Section.

### blastp

For each of the four training sets, the data were first preprocessed by constructing a blastp database on all the sequences in the training set fold. For each query sequence in the test set, blastp performed a search against the database for the appropriate training set, and returns a list of the sequences that are most similar to the query sequence. We assign the query sequence to the family of the sequence with the best bit score.

### Inclusion thresholds

We trace out multiple points on the precision-recall curve by varying the inclusion threshold of the classification. If the query sequence’s best hit failed to meet the inclusion threshold for a given method, the query sequence was left as unclassified (i.e., it was not assigned to any family). Note that all methods have heuristics that might prevent a sequence from being scored against another family if the method can determine in advance that the match will likely be poor. Thus even if the inclusion threshold is not used, some sequences might be unclassified.

In the cases of HMMER and HIPPI, we explored the use of the Pfam curated sequence gathering cutoff thresholds for each family. However, since the Pfam threshold is chosen based on full-length sequences rather than fragmentary sequences, we also examined settings for the inclusion threshold by scaling the gathering cutoff threshold by 0 % (i.e., no minimum threshold) to 100 % (i.e., the default threshold). As the HHsearch pipeline returns the probability of membership, we examined thresholds for inclusion from 25 % (i.e., accept the most probable family that has at least 25 % support) to 99 % (i.e., only accept the family if it has 99 % support for membership). As blastp returns a BLAST bit score for the best hit (note that the BLAST bit score is not comparable to the HMMER bit score), we examine fixed BLAST bit scores thresholds from a bit score of 0 (i.e., accept the best hit regardless of the bit score) to a bit score of 45 (i.e., where the drop in recall was most noticeable).

### Evaluation criteria

For each method, we examine precision and recall for the best hit. Previous research has shown that using a single HMM on a large and evolutionarily divergent data set can lead to poor downstream analyses, and by using an ensemble of HMMs (as in the SEPP [26], TIPP [6], and UPP [27] studies) the accuracy of the analyses can be improved. These two dimensions (the number of sequences in the seed alignment and the average pairwise sequence identity of the seed alignment) are therefore of particular interest to examine the performance of our methods at the subgroup level.

### HIPPI parameter selection

In order to determine the optimal parameter settings for the maximum decomposition size *X* and minimum average identity threshold *Y*, we tested different settings for these parameters on a very small subset of the Pfam database (called the “parameter selection set”), chosen as follows.

Our goal was to find families that were very similar to each other such that the different HIPPI variants might have problems assigning the query sequences to the correct family. We created a graph where each vertex represents a single Pfam family and the edges represented sufficient similarity between the families. Ten sequences from each family were chosen at random and scored against the single profile HMM for every other family. If the average bit score of the ten sequences scored against another family’s HMM is greater than 25, an edge is drawn between the two Pfam families to represent that the families were sufficiently similar. All families with degree *at least five* (i.e., that are adjacent to at least five other families) are considered well-connected and are included in the parameter selection set; all the families they are adjacent to are also included. This produced a set of 142 Pfam families that were highly connected whose sequences would allow us to test precision at each parameter value since their sequences were mutually confusable (sequences from one family would score highly to HMMs built on the other connected families).

We consider stopping rules based on a maximum decomposition size *X* of 5 or 10 (expressed as a percentage of the full dataset) and on a minimum average identity threshold *Y* of 0 or 40 (expressed as a percentage). For each stopping rule, we consider two versions of HIPPI: a conservative version (indicated using “-c") that uses the gathering cutoff threshold *g*_*i*_ for each family $\mathcal {F}_{i}$, and an aggressive version (indicated using “-a") that drops this gathering cutoff threshold requirement. Methods are labeled as HIPPI-x (X%,Y%), where “-x” is either “-c” (conservative) or “-a” (aggressive). Note that a minimum required average sequence identity threshold of 0 % would result in the same collection of profile HMMs as in UPP.

## Results and discussion

### HIPPI parameter selection

The results on the parameter selection dataset show that while methods had similar results under most conditions, the best overall results for both the aggressive and conservative versions of HIPPI used a maximum decomposition size of 10 % and a minimum average identity threshold of 40 % (i.e., HIPPI (10 %, 40 %); see Table 1). Thus, we selected 10 % as the default decomposition size and 40 % as the minimum average identity threshold; we refer to this setting as default HIPPI. When we fix the decomposition size to be 10 % and compare the impact of using a minimum average identity threshold versus not using a threshold (i.e., 40 % identity versus 0 % identity), the results show that using a minimum average identity threshold resulted in comparable or better precision (2 out of 6 cases with precision improvement of 1 % or greater, remaining four cases with precision within 1 % of each other) and better recall (5 out of 6 with recall improvement of 1 % or greater; remaining one case with recall within 1 % of each other). Prior uses of the *PP methods did not consider sequence identity as a determination for decomposition; these results show that taking the evolutionary diameter of the alignment into account results in improved accuracy.

### Running time

All HHsearch variants took at least 10 seconds per query sequence on a node with 32 cores. Actual times were often longer, but I/O issues may have contributed in some cases so an accurate benchmark was not possible. Nonetheless, this meant that a single cross-fold set of test data required over 2,500 node hours, whereas all other methods ran in less than 24 node hours. On a node with 32 cores, blastp required 13 h and 34 min to analyze a single cross-fold set, HMMER completed in 57 min and HIPPI finished in 11 h and 54 min. The running time difference between HMMER and HIPPI is not too surprising; HIPPI had 14 times more HMMs than HMMER (159,602 HMMs versus 11,156 HMMs). In terms of preprocessing time, blastp required less than a minute to build its BLAST database, HMMER required 2 min to build its profile HMMs, and HIPPI required 53 min to build its ensembles of profile HMMs, with more than 55 % of HIPPI’s time spent on building the ML trees used to generate the decompositions.

### Precision and recall

We were only able to run HHsearch on a single cross-fold due to HHsearch’s high computational costs. Thus, the data shown here for all methods are restricted to results on a single cross-fold set, which includes 312,841 query sequences of each fragment length (full-length, half-length, and quarter-length; 938,523 sequences total). For all other methods the precision and recall results were virtually identical across all four cross-fold sets (see Additional file 1: Figure A2).

Figure 3 contains precision-recall curves for each of the five different methods under consideration. The curves are estimated by varying an inclusion threshold parameter for the particular method and producing five to seven distinct points, with intermediate values interpolated linearly. Figure 4 contains additional precision-recall curves that are similar to Fig. 3, but where families are grouped according to the size of the seed alignment and its average pairwise sequence identity. The grouping by size has only two levels: families with 0 to 100 seed sequences and those with more than 100 sequences. The grouping by average pairwise sequence identity has three levels: 0–20 %, 20–30 %, and more than 30 %, which represent about 5 %, 28 % and 67 % of the 11,156 families, respectively.

Figure 3 shows that for all query sequence lengths, HIPPI dominates all other methods at every one of its computed points on the curve. Figure 4 shows that HIPPI’s improvement over the other methods is not localized to one part of the data space; the HIPPI precision-recall curve is the most outward from the origin in nearly every case, though the degree of improvement that HIPPI has over the other methods depends on the query sequence length, average pairwise sequence identity within the seed alignment, and the number of seed sequences.

For example, under the easiest conditions (full-length conditions with high sequence identity), all methods do very well, but HIPPI and HMMER are very close in performance, with the other methods having lower precision and recall. On the most fragmentary query sequences (quarter-length sequences), all methods degrade in recall (and, to a lesser extent, in precision), but HIPPI remains the most accurate with respect to both precision and recall and blastp becomes the next most accurate. Furthermore, on the most fragmentary sequences, the degree of improvement of HIPPI over the other methods is the largest.

Similarly, the query sequence length also impacts the choice of how to run the HHsearch pipeline (Fig. 3): one iteration of HHblits clearly dominates two iterations on the full-length sequences, has slightly better performance on the half-length sequences, and has slightly worse performance on the quarter-length sequences. For full-length sequences, we conjecture that the first iteration returns a sufficient number of homologs for building an HMM and that the second iteration therefore includes too many remote homologs, which in turn negatively impacts the resulting HMM. However, for shorter sequences the reverse is true: the first iteration returns fewer homologs than necessary, and a successive iteration improves results.

The differences between HIPPI and the other methods are significant. For example, the comparison between HIPPI and HMMER on the full-length sequences shows that HIPPI provides an increase in recall for the same precision of roughly 0.5 %, which is statistically significant with *p*<0.001 using a binomial test. The degree of improvement increases on the fragmentary sequences, where HIPPI provides a substantial increase in recall compared to HMMER, BLAST, and the HHsearch variants. For example, at 99.2 % precision, on half-length sequences HIPPI’s recall was 6 % greater than HMMER, 16 % greater than BLAST, and 26 % greater than the HHsearch variants, and on quarter-length sequences it is 17 % greater than HMMER, 10 % greater than BLAST, and 34 % greater than the HHsearch variants.

## Conclusion and future work

HIPPI is a new method for assigning membership of query sequences to protein families and superfamilies. When used with the Pfam database, HIPPI provides higher precision and recall in comparison to the use of a single HMM, blastp, and HHsearch. Furthermore, the improvement in precision and recall is very substantial for those protein families that have seed sequence alignments with low average pairwise sequence identity, or when the query sequence is fragmentary. Thus, HIPPI enables highly accurate family identification of amino acid sequences, even for very challenging conditions.

The key advance in HIPPI over previous ^∗^PP methods (SEPP, TIPP, and UPP) is the method used to construct the ensemble of HMMs used to represent a seed alignment. While the previous ^∗^PP methods differed in various respects in how they built their ensemble of HMMs, their decompositions used stopping rules that only considered either the number of sequences in each subset or the total number of subsets created. The dataset decomposition technique that HIPPI uses, however, also considers the average pairwise sequence identity within the subsets to determine whether to continue the decomposition. This new technique for building an ensemble of HMMs enables HIPPI to select the “best” decomposition for protein families that can have very different properties in terms of the number of sequences and in the evolutionary diameter of the family (i.e., sequence heterogeneity), and leads to improved accuracy for protein family classification compared to the ^∗^PP decomposition strategies.

The simplicity of the HIPPI approach shows that dramatic improvements in accuracy - in terms of both precision and recall - are obtainable through divide-and-conquer strategies. Thus, although we tested HIPPI only in conjunction with profile HMMs computed using HMMER, comparable improvements might be achievable for other techniques (such as the profile HMMs used within the HHsearch pipeline, position-specific profiles, support vector machines, and Markov random fields) that are used to represent protein families, subfamilies, or superfamilies.

This study also showed that the accuracy of the HHsearch pipeline with respect to protein family identification on Pfam is substantially impacted by the choice of algorithmic parameter setting (local vs. global, and the number of HHblits iterations). However, every variant of this pipeline we studied was less accurate (for both precision and recall) than HIPPI, and typically less accurate than the use of a single profile HMM (results that are consistent with [31]). Yet, since the ensemble of HMM techniques used in HIPPI is designed to improve accuracy of bioinformatics tasks that use HMMs, we conjecture that integrating the ensemble of HMM technique in HIPPI into the HHsearch pipeline could lead to even better protein family classification than HIPPI currently achieves.

The current implementation of HIPPI uses the centroid edge in the tree to partition the subsets. However, another approach that might provide improved accuracy is partitioning the tree on the longest edge in order to increase the separation between the subsets, until all the subsets are sufficiently small and homogeneous. The study evaluating variants of the UPP multiple sequence alignment method [27] showed that dividing on the longest edge resulted in no improvements in alignment accuracy, but had an increased cost of running time due to more subsets being generated. It may be the case, however, that the longest edge decomposition has better performance for protein family identification; we are currently exploring this idea.

Currently, HIPPI assigns the query sequence to the protein family that resulted in the best bit score. However, the HIPPI algorithm also returns the bit score of the query sequence scored against all the profile HMMs. It is possible to convert the bit scores into confidence scores (i.e., the probability of a protein family for generating the query sequence) using the alignment support calculation equations provided in [6]. We plan to include confidence scores in future versions of HIPPI.

Given the improvement obtained using the new dataset decomposition strategy, we conjecture that modifications to the dataset decomposition strategies used in the previous ^∗^PP methods could lead to improvements for their respective tasks. For example, one of the key tasks in TIPP [6] is the assignment of metagenomic shotgun data to marker genes. HIPPI’s superior accuracy on fragmentary sequences may lead to improved abundance profiles by enabling more accurate assignments of the fragmentary reads to protein families or gene families, and could result in improvements to other marker-based profiling methods such as mOTU [32], MetaPhyler [4], and MetaPhlAn [5].

However, other design strategies may provide even better advantages. Indeed, the overall observation in this study, as well as in [3, 6, 20, 23, 24, 26, 27], is that representations of multiple sequence alignments by ensembles of HMMs (however constructed) provide improved accuracy for many bioinformatics tasks compared to the use of a single HMM, and are often better than the leading alternative methods. Thus, perhaps the design strategies used by [3, 20] will provide even better accuracy. Further research is needed to find the best ways of constructing and using ensembles of HMMs, while also providing highly efficient and easy to use implementations.

## Additional file

Additional file 1 HIPPI Supplement. Additional file 1 contains the commands used to generate the results presented in the paper, Fig. A1 showing the results of different variants of the HHsearch pipelines, and Fig. A2 showing the results of HIPPI, HMMER, and BLAST evaluated on all four cross-folds subsets of the data. (PDF 139 KB)

## Abbreviations

HMM: Profile hidden Markov model
HIPPI: Hierarchical profile HMMs for protein family identification
ML: Maximum likelihood
