# Supplementary material for: HIPPI: highly accurate protein family classification with ensembles of HMMs
Source: BMC Genomics. 2016 Nov 11;17(Suppl 10):765. doi: 10.1186/s12864-016-3097-0 (PMC5123343; doi:10.1186/s12864-016-3097-0)
Supplement: Supplementary file 1 — HIPPI Supplement. Additional file 1 contains the commands used to generate the results presented in the paper, Fig. A1 showing the results of different variants of the HHsearch pipelines, and Fig. A2 showing the results of HIPPI, HMMER, and BLAST evaluated on all four cross-folds subsets of the data. (PDF 139 KB) [file 12864_2016_3097_MOESM1_ESM.pdf]

HIPPI Supplement

Nam-phuong Nguyen[1], Mike Nute[2], Siavash Mirarab[3], and Tandy Warnow[2*]

[1]Computer Science and Engineering, University of California, San Diego,
[2]Department of Computer Science, University of Illinois at Urbana-Champaign,
[3]Department of Electrical and Computer Engineering, University of California, San Diego,
[*]To whom correspondence should be addressed; E-mail: warnow@illinois.edu.

August 17, 2016

Contents

List of Figures

A1 **Precision-recall curves for three parameter settings tested for the HH-search pipeline.** The curves are estimated by varying an inclusion threshold parameter for the particular method and producing five to seven distinct points, with intermediate values interpolated linearly. **1 Iter.** and **2 Iter.** each refer to the default HHsearch settings (local alignment) paired with HHblits using 1 and 2 iterations, respectively. **2 Iter. Global** refers to the default HHblits settings (2 iterations) paired with HHsearch using the global alignment option. 5

A2 **Precision-recall curves for HIPPI, HMMER, and blastp, evaluated on all four cross-fold subsets of the data.** The curves are estimated by varying an inclusion threshold parameter for the particular method and producing five to seven distinct points, with intermediate values interpolated linearly. Note that the scales for both axes vary between panels due to the significant impact of sequence fragmentation. 6

Commands

To run blastp [1], we built a BLAST database on the set of sequences in the training set. We then scored the test set against the BLAST database using blastp. All commands were run using BLAST 2.2.31+ and are given below.

- **Building BLAST database**: *makeblastdb -in <input_sequences> -dbtype prot*
- **BLAST search**: *blastp -db <blast_database> -out <output> -query <query_sequences> -num_threads 32 -outfmt 6*

The HMMs used by HMMER [2, 3] and all variants of HIPPI were generated using a python script called "ensemble.py" that is available on GitHub at *https://github.com/smirarab/sepp*. The module acts as a wrapper around the HMMER suite of tools and builds an ensemble of HMM profiles from a given alignment and tree. The module takes as input an alignment and tree, and the stopping parameters. The HMMER HMM profiles were collected into a single HMM file and then the query sequences were scored against the HMMER HMM profile file using hmmsearch. All HMMER commands were run using HMMER version 3.1b2. The ML tree was estimated using FastTree-2 version 2.1.7 [4].

We provide the commands for each method below, as well as the internal calls to HMMER.

- **FastTree-2**: *fasttreeMP <input_alignment> > <output_fasttree>*
- **hmmbuild**: *hmmbuild –cpu 1 –amino –informat afa <output_hmm_model> <input_alignment>*
- **hmmsearch**: *hmmsearch –noali –cpu 1 <input_hmm_model> <query_sequences>*
- **HMMER/HIPPI**: *run_ensembles.py -x 32 -a <input_alignment> -t <input_fasttree> -f <query_sequences> -A <max_decomp_size> -m amino -D <maximum_dissimilarity>*

where *max_decomp_size* is equal to the number of sequences in the input alignment (for HMMER) or to a percentage of the number of sequences (for HIPPI), and *maximum_dissimilarity* is set to 0.60 if we require a 40% sequence identity to stop decomposing.

We provide the commands for running the HHsearch-HHpred pipeline [5] below. To run the pipeline we first ran each query sequence against the UniProt20 database using HHblits. We then convert the HHblits results into a FASTA alignment file using the hhr2fas.py python script. In the event that the HHblits output contains no hits (i.e., zero homologies to any sequences within the UniProt20 database), the hhr2fas.py script returns an empty alignment; in this case we replace the empty alignment with an alignment containing only the query sequence. Next, we built HMMs on each Pfam seed alignment using HHmake. We compile all the HMMs into an HMM database using hhsuitedb.py. Finally, we scored the quality of the HMM-HMM alignment of the HMMs created from the HHblits results against the HMM database using HHsearch. All programs and scripts listed below are from the HH-suite software version 3.0.0, with the exception of the script hhr2fas.py, which was a custom script provided to us by the authors of HH-Suite.

- **HHblits**: *hhblits -i <query_sequence> -o <hhblits_output> -n [1—2] -d uniprot20_2015_06*
- **Converting HHBlits result to FASTA**: *python hhr2fas.py <hhblits_input> <output_alignment>.*
- **Building HMM**: *hhmake -i <input_alignment> -o <output_alignment> -M 50*
- **Building HMM database**: *hhsuitedb.py –ihhm=<input_hmms> –ia3m=<input_alignments> -o <output_name> –cpu=32*
- **HHsearch**: *hhsearch -i <hhblits_result> -cpu 32 -d <pfam_database> -Z 20000 -p 0 -M*

first -all [-loc—-glob] -scores <output_scores>

Additional Figures

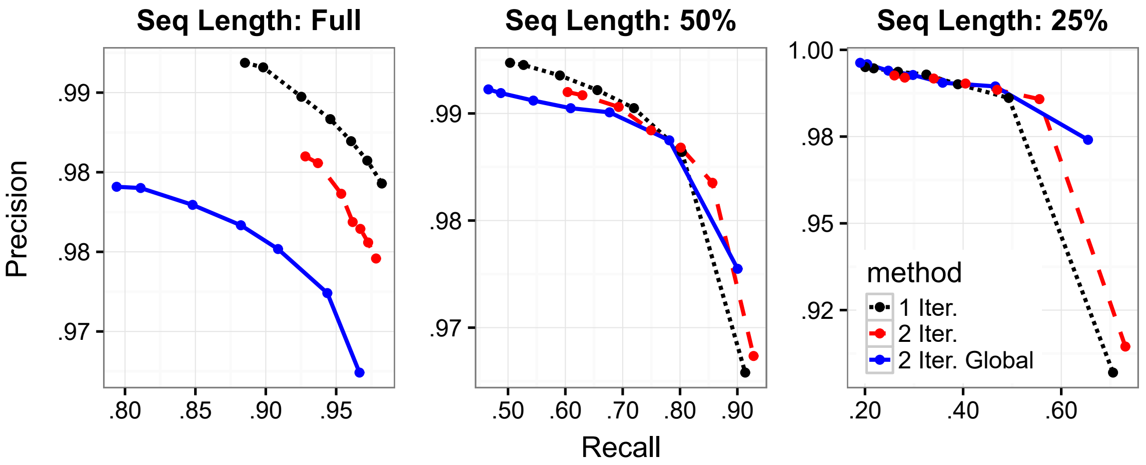

Figure A1: **Precision-recall curves for three parameter settings tested for the HHsearch pipeline.** The curves are estimated by varying an inclusion threshold parameter for the particular method and producing five to seven distinct points, with intermediate values interpolated linearly. **1 Iter.** and **2 Iter.** each refer to the default HHsearch settings (local alignment) paired with HHblits using 1 and 2 iterations, respectively. **2 Iter. Global** refers to the default HHblits settings (2 iterations) paired with HHsearch using the global alignment option.

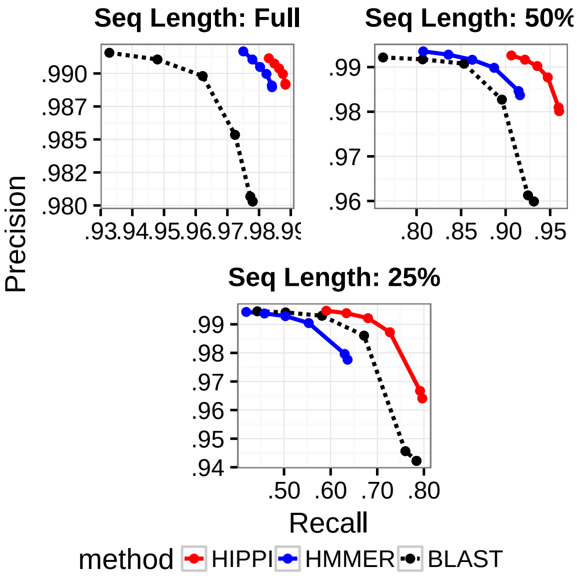

Figure A2: **Precision-recall curves for HIPPI, HMMER, and blastp, evaluated on all four cross-fold subsets of the data.** The curves are estimated by varying an inclusion threshold parameter for the particular method and producing five to seven distinct points, with intermediate values interpolated linearly. Note that the scales for both axes vary between panels due to the significant impact of sequence fragmentation.

References

[1] Altschul, S.F., Madden, T.L., Schäffer, A.A., Zhang, J., Zhang, Z., Miller, W., Lipman, D.J.: Gapped BLAST and PSI-BLAST: a new generation of protein database search programs. Nucleic Acids Res. **25**(17), 3389–3402 (1997). doi:10.1093/nar/25.17.3389. http://nar.oxfordjournals.org/content/25/17/3389.full.pdf+html

[2] Finn, R.D., Clements, J., Eddy, S.R.: HMMER web server: interactive sequence similarity searching. Nucleic Acids Res. **39**, 29–37 (2011)

[3] Eddy, S.R.: A new generation of homology search tools based on probabilistic inference. Genome Inform **23**, 205–211 (2009)

[4] Price, M.N., Dehal, P.S., Arkin, A.P.: FastTree 2–approximately maximum-likelihood trees for large alignments. PLOS ONE **5**(3), 9490 (2010). doi:10.1371/journal.pone.0009490

[5] Remmert, M., Biegert, A., Hauser, A., Söding, J.: HHblits: lightning-fast iterative protein sequence searching by HMM-HMM alignment. Nat. Methods **9**(2), 173–175 (2012). doi:10.1038/nmeth.1818
